# Societal and economic burden of migraine in Spain: results from the 2020 National Health and Wellness Survey

**DOI:** 10.1186/s10194-024-01740-3

**Published:** 2024-03-15

**Authors:** David García-Azorín, Carlota Moya-Alarcón, Beatriz Armada, Margarita Sánchez del Río

**Affiliations:** 1 Headache Unit, Department of Neurology, Hospital Clínico Universitario de Valladolid, Valladolid Health Research Institute (VALLHRI), Calle Rondilla Sta.Teresa, S/N, Valladolid, 47010 Spain; 2 Pfizer S.L.U., Madrid, Spain; 3 Department of Neurology, Clínica Universidad de Navarra, Madrid, Spain

**Keywords:** Headache disorders, Cost of illness, Depression, Public health, Nervous system diseases

## Abstract

**Background:**

The burden of migraine goes beyond the pain and associated symptoms. We aimed to describe the impact of migraine in healthcare resource utilization (HCRU), work productivity, and mood disorders, as well as its economic cost.

**Methods:**

Case–control study nested in a cross-sectional analysis of patient-reported data collected between 30/12/2019 and 20/04/2020 as part of the National Health and Wellness Survey, from respondents located in Spain. Adults (≥ 18 years old) who reported a physician diagnosis of migraine and ≥ 1 monthly headache days (MHD) in the previous 30 days were included. HCRU, health-related quality-of-life, depression scores, work and activity impairment, and the associated direct and indirect costs were assessed for four cohorts of migraine patients, according to the frequency of headache (MHD: 1–3, 4–7, 8–14, ≥ 15) and compared to a no-migraine control, matched to migraine cases by a propensity score based on demographic and clinical variables.

**Results:**

The survey was completed by 595 people with active migraine, of whom 461 (77.4%) experienced < 8 MHDs and 134 (22.6%) ≥ 8 MHDs, and 1,190 non-migraine matched controls. Migraine patients presented worse mental and physical health functioning (SF-12 MCS: 41.9 vs. 44.7, *p* < 0.001; SF-12 PCS: 48.6 vs. 51.5, *p* < 0.001), worse self-reported health (EQ-5D VAS: 65.8 vs. 73.5, *p* < 0.001), more severe depression (PHQ-9: 8.9 vs. 6.1, *p* < 0.001), and higher overall work impairment (WPAI: 41.4 vs. 25.5, *p* < 0.001). People with migraine had higher HCRU, twice higher hospitalization rates (17.0% vs. 8.3%, *p* < 0.001) and 1.6 higher emergency room (ER) visit rates (51.4% vs. 31.2%, *p* < 0.001). Having migraine translated into higher annual costs with HCRU (€894 vs. €530) and productivity losses (€8,000 vs. €4,780) per person. Respondents with more MHDs presented worse outcomes and higher costs but suffering from 1–3 MHD also increased costs by 51.3%.

**Conclusions:**

Having migraine not only causes a massive impact on patients’ quality of life and ability to work, but it also generates considerable economic costs for society. In Spain, having migraine was associated to 1.7 higher costs per patient. The clinical and economic burden increases with the frequency of headaches but is higher than controls even in patients suffering from 1–3 MHD.

**Graphical Abstract:**

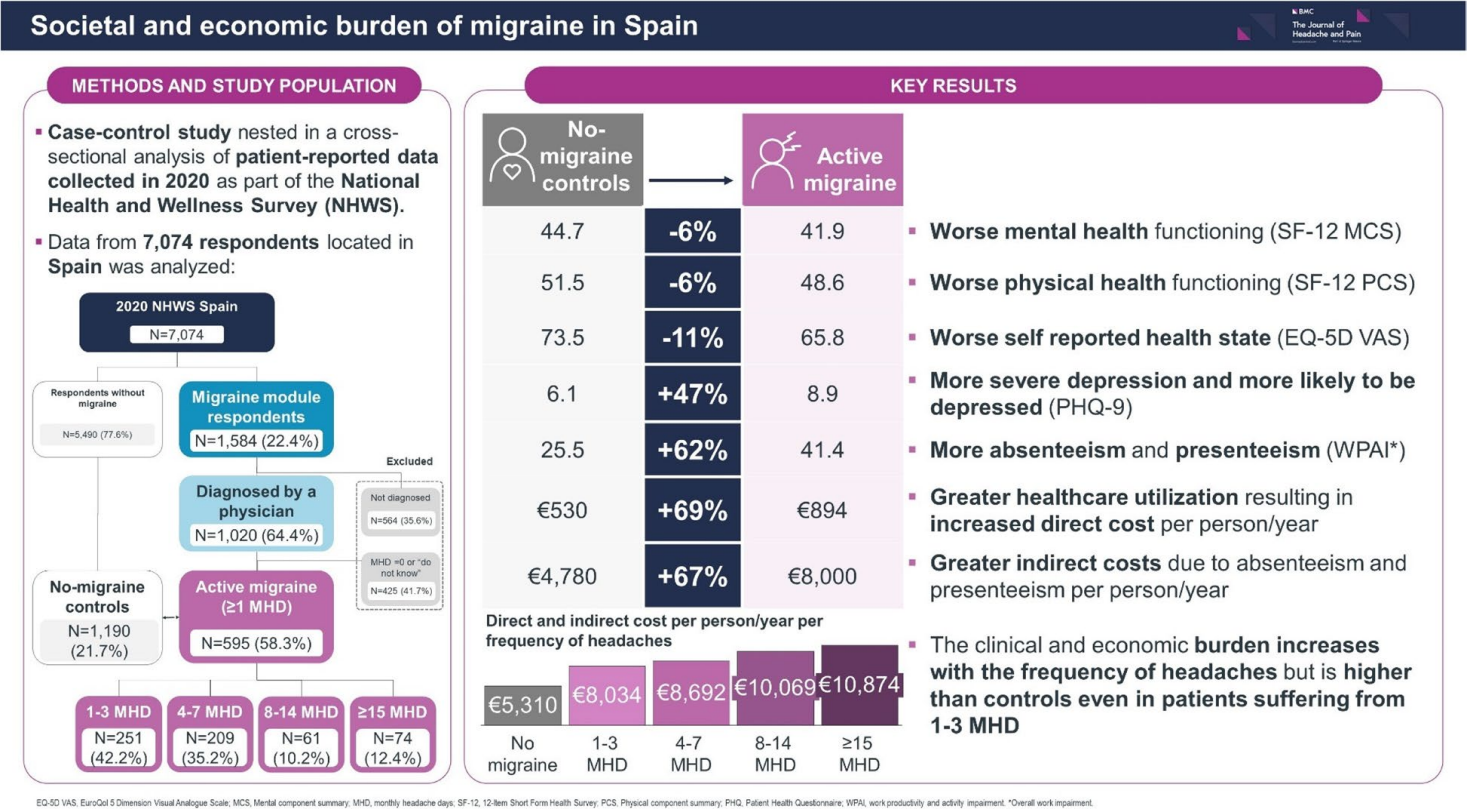

**Supplementary Information:**

The online version contains supplementary material available at 10.1186/s10194-024-01740-3.

## Main text

### Background

Migraine is a neurologic disease ranked as the second-highest cause of disability worldwide, and the highest among women between 15–49 years of age [1]. In Spain, the 1-year prevalence was estimated at 12.6%. Despite its high prevalence and impact on daily life, migraine is still an underdiagnosed and undertreated medical condition [2–4].

Migraine can be devastating, impacting the patients’ quality of life, affecting their ability to perform daily activities, and to work. As a result, it leads to significant direct and indirect economic costs [5–12]. In Spain, the yearly cost attributed to migraine per patient is roughly eleven thousand euros, with 60–70% stemming from indirect costs due to lost productivity [3, 10, 13]. The impact of migraine is notably greater in individuals with chronic migraine (CM) and those experiencing more frequent headaches and a poorer response to preventive treatments. The 2018 Spanish Atlas of migraine revealed that the total yearly cost for a patient with CM was 2.6 times greater than for one with episodic migraine (EM) [3, 10]. Nonetheless, patients with less frequent headache episodes are also affected in their everyday tasks. While the focus of most research has been on patients with CM or high-frequency EM, the vast majority of people living with migraine experience fewer monthly headache episodes [7].

The present study aimed to bridge this gap, by examining the societal and economic impact of migraine according to the frequency of headaches – in monthly headache days (MHD) – with respect to people without migraine. The study measures and compares the Healthcare Resource Utilization (HCRU), Health Related Quality of Life (HRQoL), work productivity and activity impairment (WPAI), and direct and indirect costs of migraine across different population cohorts.

### Methods

#### Study design

This study was a case–control study, nested in a cross-sectional analysis of patient-reported data collected in 2020 as part of the National Health and Wellness Survey (NHWS, Cerner Enviza) from respondents located in Spain.

#### Study setting and period

The NHWS is a self-administered, internet-based survey conducted in several countries. The survey consists of a base questionnaire that assesses demographics and health characteristics, and disease-state specific modules that are only completed by respondents who self-reported a diagnosis of the disease state [14]. Potential respondents were identified primarily through participation in opt-in online survey panels, with stratified quota sampling to ensure country-specific representation in terms of age and gender. In Spain, online panel recruitment was complemented by computer-assisted web interviews in which respondents were recruited via telephone and could choose to complete the interview either on the phone, on a computer in a private center, or through an emailed link [14].

The 2020 Spanish NHWS was conducted between 30 December 2019 and 20 April 2020, using a physician reviewed Spanish-language survey version.

#### Study population

All respondents were residents of Spain, aged 18 years or older, that consented to participate in the survey, and spoke Spanish as primary language [14].

#### Active migraine group

In this analysis, cases were respondents who: 1) reported a physician diagnosis of migraine, and 2) had ≥ 1 MHD in the past 30 days. Respondents were excluded if they did not have a physician diagnosis of migraine, they “did not know” or reported no MHD in the previous 30 days.

#### No migraine control group

People without migraine were included as “no migraine” and were matched to people with migraine by propensity scores using eleven sociodemographic characteristics.

#### Data sources / measurements

The NHWS included demographic variables, general health characteristics, health status, experienced and/or diagnosed comorbidities, depression and anxiety symptoms, work productivity, and healthcare resource utilization (HCRU). All information was self-reported by respondents. No clinical charts were reviewed [15].

### Outcomes

#### Demographic and clinical characteristics

Demographic and general health and clinical variables included age, sex, marital status, education, household income, employment status, body mass index (BMI), smoking behavior, alcohol use, and exercise behavior. The Charlson Comorbidity Index (CCI)—a weighted index to predict risk of death within ten-years for patients with specific comorbid condition—was also calculated. In CCI, the results may vary between 0 and 24, where the higher the score, the higher the risk of death [16]. Migraine patients were stratified into four subgroups, according to the mean number of monthly headache days in the past 30 days (MHD: 1–3, 4–7, 8–14, ≥ 15). The full list of study variables and definitions is available as supplementary material.

#### Health‑Related Quality of Life (HRQoL)

The study used two validated instruments to assess patient-reported HRQoL, namely the 12-Item Short Form Survey Instrument (SF-12) and the EuroQuol-5 dimensions (EQ-5D) [17–19].

The SF-12 survey evaluates the general health status through eight health domains, assessing both physical and mental health, through two summary scores: the physical component summary (PCS-12) and the mental component summary (MCS-12). The score ranges from 0 to 100, with a higher score indicating better physical and mental health functioning. Scores ≤ 50 on the PCS-12 may be indicative of a physical condition and ≤ 42 on the MCS-12 indicative of 'clinical depression’ [17–20].

The EQ-5D was used as a self-report measure of health. Five dimensions are assessed (mobility, self-care, usual activities, pain/discomfort, and anxiety/ depression) to generate the EQ-5D Utility Index, which ranges between zero (‘a state as bad as being dead’) and one (‘full health’). The EQ-5D visual analog scale (EQ-VAS) was also used, where respondents are asked to linearly score their self-rated health with the possible results ranging between zero (‘worst imaginable health state’) and 100 (‘best imaginable health state’) [19].

#### Depression

Patient Health Questionnaire (PHQ-9) was used to screen for depression. It measures the frequency of depression symptoms, generating a score that ranges from 0 to 27 (0–4, indicates none or minimal depression; 5–9, mild depression; 10–14, moderate depression; 15–19 moderate-to-severe depression, and; 20–27 severe depression) [21].

#### Work productivity and activity impairment

Work productivity and activity impairment (WPAI) were assessed using the General Health version of the WPAI questionnaire (WPAI-GH), a 6-item validated instrument with four metrics: activity impairment, absenteeism, presenteeism, and overall work impairment. All respondents provided data for activity impairment (the percentage of impairment in daily activities because of one’s health in the past 7 days). Only respondents who reported being full-time, part-time, or self-employed provided data for absenteeism (the percentage of work time missed because of one’s health in the past 7 days), presenteeism (the percentage of impairment experienced while at work in the past 7 days because of one’s health), and overall work productivity loss (an overall impairment estimate that is a combination of absenteeism and presenteeism). All metrics are expressed as impairment percentages, ranging from zero to 100, with higher numbers indicating greater impairment and lower productivity [14].

#### Healthcare resource utilization

Respondents were asked about HCRU in the past 6 months, specifically: primary care visits, visits to the neurologist, emergency room (ER) visits and hospitalizations. The mean number of visits for each provider type and care setting in the past 6 months were recorded. Reported HCRU values were multiplied by two, to provide estimates for a complete year, assuming a constant HCRU.

#### Direct and indirect economic burden

Direct healthcare cost associated with HCRU were computed by multiplying the number of visits by their estimated unit cost in Spain, as detailed in supplementary materials. The unit costs used by Irimia et al. (2022) [22] – based on data from the healthcare centers’ participating in the PERSEC study, conducted in 2019 – were updated to 2023 values, adjusting for inflation [23].

Indirect costs associated to lost work productivity were estimated from NHWS data using the human capital method [14]. Income statistics per gender and quartiles for Spain were retrieved from the National Institute of Statistics for 2021 [24] and inflated to 2023 values [23]. NHWS data on the income distribution, per gender, of migraine and no-migraine respondents (below median income [%], at median income [%], above median income [%]) was used to estimate mean annual household incomes for the study population. A mean annual household income of €27,072 was considered for the active migraine group and of €27,006 for the no-migraine control group. Annual incomes were then multiplied by the overall work productivity loss reported by each group to represent societal lost earnings per employee per year for absenteeism and presenteeism. This cost was weighted by the employed population in each group to compute a mean annual indirect cost per person in each group, as detailed in supplementary materials.

#### Statistical analysis

There was no formal sample size estimation, and all the analyses were conducted in the available data.

#### Matching to no-migraine comparisons

Matching was conducted by generating a propensity score of having active migraine vs. not having migraine based on eleven demographic and clinical characteristics. Each person with migraine was matched with two respondents without migraine (a 1:2, case:control ratio). The propensity score was estimated using logistic regression predicting the presence of migraine for each person included in the study sample (no-migraine comparisons and those with MHD ≥ 1). The model included demographic (age, sex, marital status, household income, level of education), health (alcohol use, vigorous exercise, BMI) and clinical (CCI) measures. Cardiovascular diseases were not included. Matching was conducted using a greedy matching algorithm [25] that first required an exact match and then matched on the propensity score within a caliper of ± 0.1 standard deviations of the mean propensity score. If a match was not found for a person with migraine, they were excluded from further analyses.

#### Statistical analysis

##### Comparison of respondents with and without migraine

Demographic, health, and clinical characteristics were summarized by means and standard deviations for continuous measures (age, CCI) and counts and percentages for categorical variables. Arithmetic means were selected to report results as they provide the information required for healthcare policy decisions, by informing about the total cost that will be incurred by treating all patients [26]. Comparisons of these characteristics for both the pre-matched and post-matched samples used standardized mean differences, where a difference ≥ 0.10 (10%) was interpreted as a meaningful difference between those with and without migraines. Active migraine cases were stratified by MHDs experienced during the prior month. Comparisons of HRQoL, HCRU, and WPAI measures were conducted for the overall active migraine group and by categories of MHD (1–3, 4–7, 8–14, ≥ 15) versus their matched no-migraine comparators. Bivariate analyses were conducted to illustrate differences between cohorts, with significance determined by a *p*-value < 0.05. HCRU was compared using McNemar’s test for any use and independent samples paired t-tests for comparison of group means. SF-12 MCS and PCS scores, EQ-5D utilities, EQ-VAS, PHQ-9, and WPAI summary scores comparisons used independent samples paired t-tests. PHQ-9 depression categories were compared with McNemar’s test.

##### Missing data

No methodology was necessary to account for missing data as NHWS was an opt-in online survey that did not allow for missing responses, therefore only including respondents who provided complete data.

##### Ethical considerations

The study was conducted following the ethical principles of the Declaration of Helsinki and the local regulation, including privacy laws. The 2020 NHWS data were reviewed and granted exemption status by the Pearl Institutional Review Board (Indianapolis, IN).

### Results

#### Demographic and clinical characteristics

**Fig. 1:**
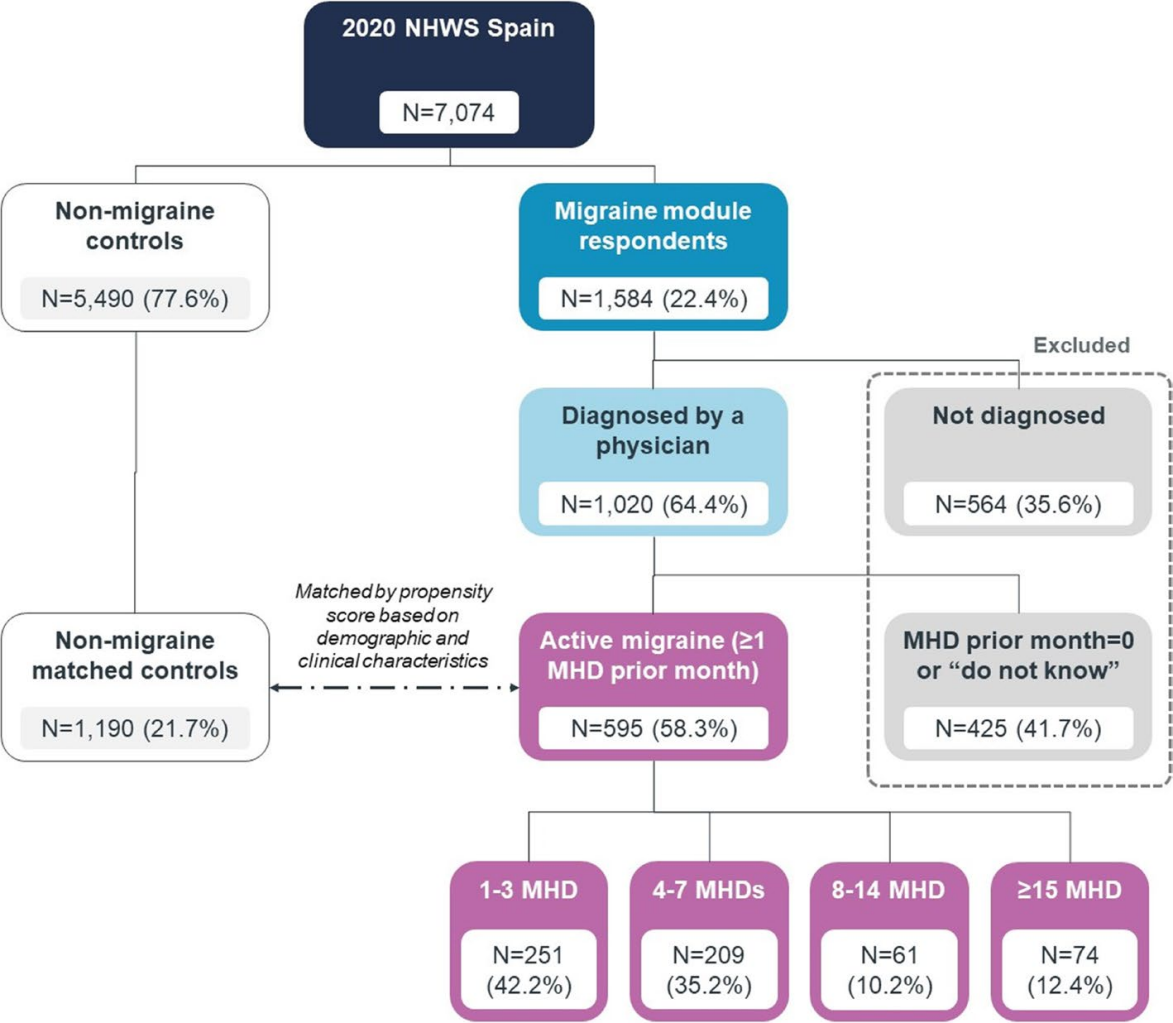
Flow chart of the study population. MHDs, monthly headache days; NHWS, National Health and Wellness Survey

The NHWS survey was completed by 7,074 respondents in Spain, of whom 1,584 (22.4%) reported experiencing migraine in the past 12 months and completed the migraine module with additional questions on migraine characteristics. Of these respondents, 1,020 (14.4%) reported a physician-diagnosed migraine, and 595 (8.4%) of them had ≥ 1 MHD in the past 30 days and were classified as active migraine cases (Fig. 1). Within active migraine cases, 461 (77.4%) experienced < 8 MHDs and 134 (22.6%) experienced ≥ 8 MHD. Cases were matched with 1,190 no-migraine controls.

Mean age was 41.2 (12.1 SD) years in the active migraine group, with women accounting for 400/595 (67.2%) cases. Across both groups, most respondents reported earnings above median income (41–42%) and were employed (69–71%). All demographic and clinical characteristics are shown in Table 1.

**Table 1:** Demographic and clinical characteristics of people with active migraine versus no-migraine matched controls

**Characteristics**		**No migraine**^a^ **(*n* = 1,190)**	**Active migraine [≥ 1MHD] (*n* = 595)**
Age (years)	Mean (SD)	40.9 (13.4)	41.2 (12.1)
Sex	Male (%)	374 (31.4)	195 (32.8)
	Female (%)	816 (68.6)	400 (67.2)
Marital status	Single/not living with partner (%)	335 (28.2)	162 (27.2)
	Married/living with partner (%)	855 (71.9)	433 (72.8)
	Decline to answer (%)	0 (0.0)	0 (0.0)
University education	Less than University education (%)	704 (59.2)	351 (59.0)
	University education or higher (%)	480 (40.3)	242 (40.7)
	Decline to answer (%)	6 (0.5)	2 (0.3)
Annual household income	Below median income (%)	360 (30.3)	184 (30.9)
	Median income (%)	281 (23.6)	134 (22.5)
	Above median income (%)	493 (41.4)	252 (42.4)
	Decline to answer (%)	56 (4.7)	25 (4.2)
Employed (FT/PT/SE)	Yes (%)	825 (69.3)	425 (71.4)
	No (%)	365 (30.7)	170 (28.6)
CCI	Mean (SD)^b^	0.5 (1.2)	0.6 (1.1)
BMI	Mean (SD)	24.9 (6.7)	24.7 (6.7)
Alcohol use	Do not drink (%)	319 (26.8)	155 (26.1)
	Drink alcohol (%)	871 (73.2)	440 (74.0)
Smoking behaviour	Never smoked (%)	379 (31.9)	190 (31.9)
	Former smoker (%)	280 (23.5)	142 (23.9)
	Current smoker (%)	531 (44.6)	263 (44.2)
Exercise	Mean number of days/month (SD)	8.2 (8.9)	8.3 (8.1)

*BMI* Body Mass Index, *CCI* Charlson Comorbidity Index, *FT* Full-time, *MHD* Monthly headache days, *SD* Standard Deviation, *SE* Self-employed, *PT* Part-time

^a^Matched controls

^b^Including zero

#### Health‑related quality of life

**Fig. 2:**
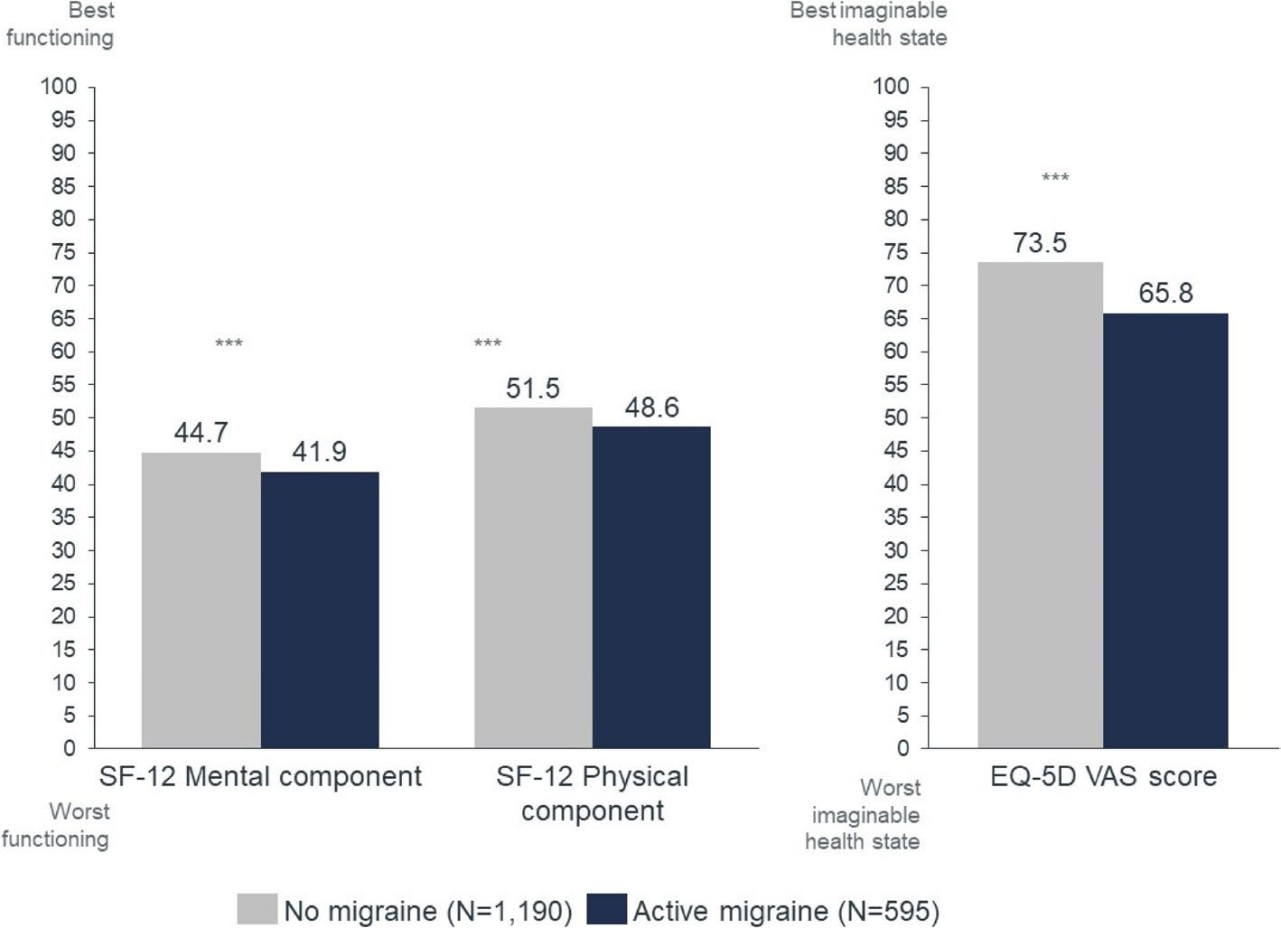
Mean score comparison of the patient reported outcomes in SF-12 health survey and EQ-5D questionnaire in active migraine vs. no migraine. EQ-5D VAS, EuroQol 5 Dimension Visual Analogue Scale; SF-12, 12-Item Short Form Health Survey. *** *P* < 0.001

Analyses revealed significant differences (*p* < 0.001) between the two cohorts in SF-12 and EQ-5D (Table 2). Patients with active migraine reported worse mean SF-12 mental component summary scores (41.9 vs. 44.7), worse SF-12 physical component scores (48.6 vs. 51.5), and worse health state – measured by the EQ-VAS – (65.8 vs. 73.5) than the no-migraine group (Fig. 2). Migraine’s negative impact on HRQoL increased with the monthly frequency of headaches reported by patients (Table 2).

**Table 2:** Comparison of quality of life, depression, productivity, and healthcare resource utilization across cohorts

**Parameter**	**No migraine**^a^ **(*n* = 1,190)**	**1–3 MHD (*N* = 249)**	**4–7 MHD (*N* = 176)**	**8–14 MHD (*N* = 93)**	** ≥ 15 MHD (*N* = 77)**
**Health-related quality of life**					
SF-12 MCS, mean (SD)	44.7 (9.4)	43.4 (9.5)^*^	42.0 (7.9)^***^	40.4 (6.3)^***^	38.4 (10.8)^***^
SF-12 PCS, mean (SD)	51.5 (8.1)	49.8 (8.0)^**^	48.8 (8.5)^***^	47.2 (8.3)^***^	46.0 (9.7)^***^
EQ-5D utility score, mean (SD)	0.8 (0.2)	0.8 (0.2)^*^	0.8 (0.2)^***^	0.7 (0.2)^***^	0.7 (0.3)^***^
EQ-5D VAS score, mean (SD)	73.5 (23.2)	70.8 (22.7)	65.5 (25.9)^***^	63.8 (23.4)^***^	52.9 (27.8)^***^
**Depression**					
PHQ-9 score, mean (SD)	6.1 (5.8)	7.8 (6.3)^***^	8.8 (6.5)^***^	10.0 (5.8)^***^	11.8 (7.3)^***^
PHQ-9 score segment, n(%)					
None-minimal depression (0–4)	589 (49.5)	91 (36.6)^**^	56 (31.8)^***^	16 (17.2)^***^	13 (16.9)^***^
Mild depression (5–9)	328 (27.6)	84 (33.7)^**^	53 (30.1)^***^	34 (36.6)^***^	20 (26.0)^***^
Moderate depression (10–14)	155 (13.0)	40 (16.1)^**^	34 (19.3)^***^	23 (24.7)^***^	19 (24.7)^***^
Moderately severe depression (15–19)	76 (6.4)	20 (8.0)^**^	18 (10.2)^***^	16 (17.2)^***^	11 (14.3)^***^
Severe depression (20–27)	42 (3.5)	14 (5.6)^**^	15 (8.5)^***^	4 (4.3)^***^	14 (18.2)^***^
**Productivity**					
Absenteeism score^b^, mean (SD)	7.1 (18.1)	10.5 (20.6)^*^	10.9 (19.6)^*^	11.5 (16.5)	18.7 (28.2)^***^
Presenteeism score^b^, mean (SD)	21.9 (27.3)	33.4 (29.3)^***^	34.8 (28.4)^***^	42.8 (30.2)^***^	41.9 (27.7)^***^
Total work productivity impairment score^b^, mean (SD)	25.5 (31.0)	38.3 (31.8)^***^	40.1 (31.4)^***^	46.0 (31.6)^***^	49.6 (32.5)^***^
Activity impairment score, mean (SD)	25.8 (29.0)	33.3 (27.7)^***^	38.1 (29.3)^***^	47.4 (27.7)^***^	49.7 (29.9)^***^
**Healthcare resource utilization**					
GP visits in past 6 months^c^, mean (SD)	1.5 (2.2)	1.9 (2.2)^*^	2.6 (3.1)^***^	2.8 (2.7)^***^	3.3 (3.6)^***^
Neurologist visits in past 6 months^c^, mean (SD)	0.1 (0.3)	0.1 (0.3)	0.2 (0.5)^***^	0.3 (1.1)^***^	0.6 (1.1)^***^
ER visits in past 6 months^c^, mean (SD)	0.7 (1.6)	1.0 (1.6)^*^	1.5 (2.1)^***^	1.7 (2.3)^***^	2.3 (4.5)^***^
Hospitalizations in past 6 months^c^, mean (SD)	0.2 (2.1)	0.2 (0.9)	0.3 (0.9)	0.5 (1.3)	0.4 (0.9)

*EQ-5D VAS* EuroQol 5 Dimension Visual Analogue Scale, *ER* Emergency Room, *GP* General Practitioner, *MCS* Mental component summary, *MHD* Monthly headache days, *SD* Standard Deviation, *SF-12* 12-Item Short Form Health Survey, *PCS* Physical component summary, *PHQ* Patient Health Questionnaire

Statistical significance versus no migraine matched controls: ns *P* > 0.05; * *P* ≤ 0.05; ** *P* ≤ 0.01; *** *P* ≤ 0.001

^a^Matched controls

^b^Only respondents from employed sample are included

^c^Including patients reporting zero visits

#### Depression

**Fig. 3:**
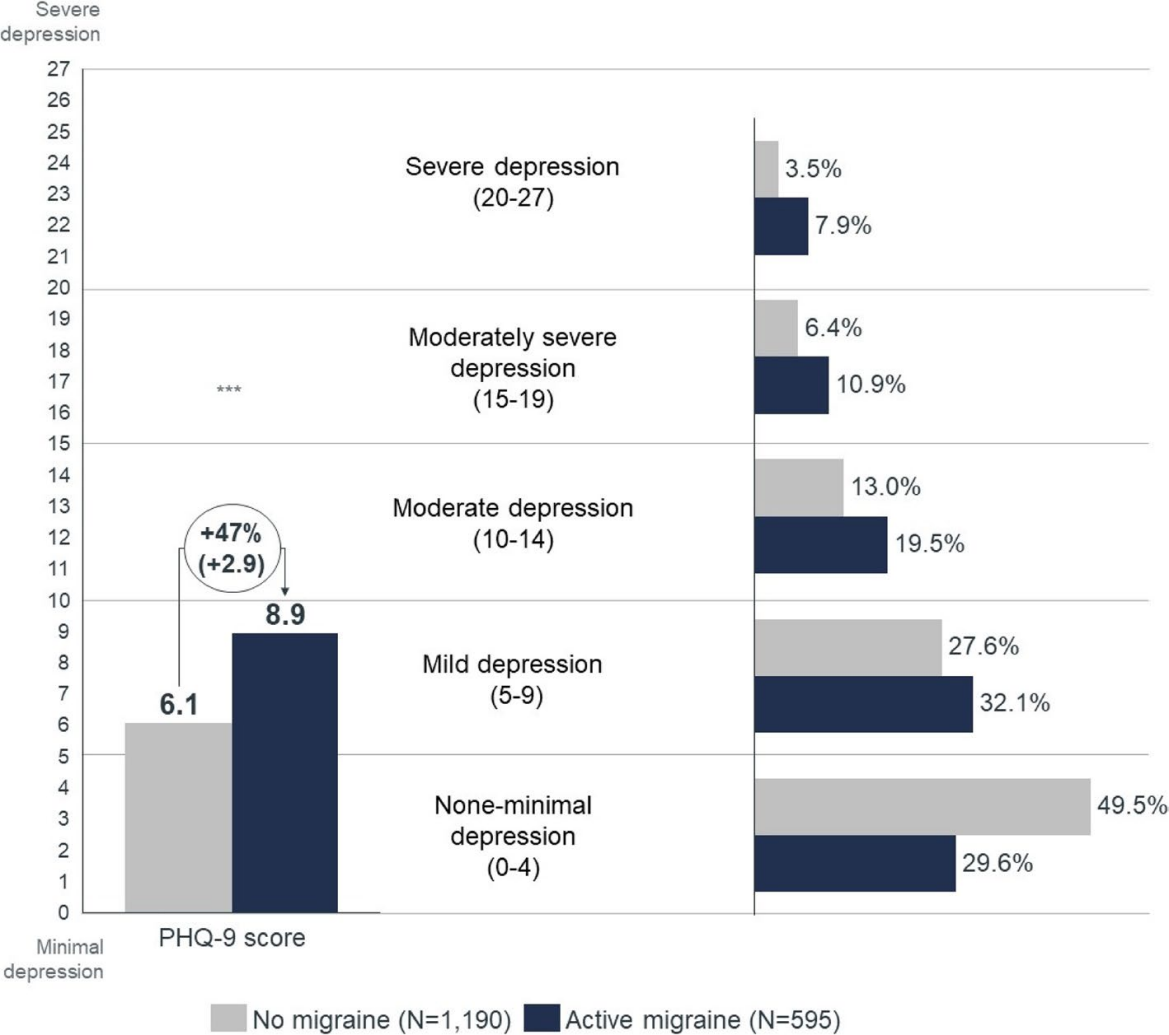
Comparison of the patient health questionnaire PHQ-9 mean score and distribution per score segment in active migraine vs. no migraine. PHQ, Patient Health Questionnaire. *** *P* < 0.001

Patients with active migraine scored higher on the PHQ-9 than the no-migraine group (8.9 vs. 6.1, *p* < 0.001) and were more likely to suffer from depression than patients without migraine (Fig. 3). All migraine patients reported worse PHQ-9 scores than no-migraine controls, and patients with higher number of MHD had worse scores (Table 2). Amongst patients with ≥ 15 MHD 18.2% presented severe depression scores, compared to 3.5% observed in those without migraine (*p* < 0.001).

#### Work productivity and activity impairment

**Fig. 4:**
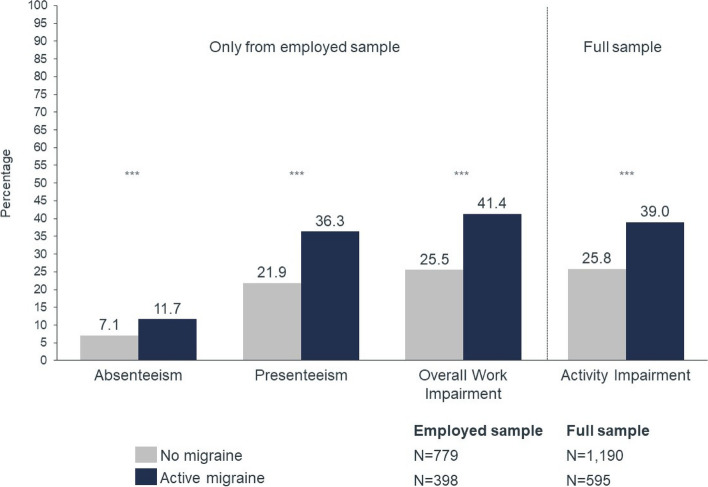
Comparison of work productivity and activity impairment metrics in active migraine vs. no migraine cohorts. *** *P* < 0.001

Work productivity and activity impairment were greater in the active migraine group than in the no-migraine control across all metrics (Fig. 4, *p* < 0.001), increasing with the frequency of headaches (Table 2). Absenteeism was 64.8% higher in individuals with active migraine (11.7% vs. 7.1%, *p* < 0.001), and more than doubled in individuals with ≥ 15 MHD (18.7% vs. 7.1%, *p* < 0.001).

#### Healthcare resource utilization

Statistically significant differences were observed in the percentage of individuals who visited each type of resources at least once in the past 6 months. Respectively, in the past 6 months: 74.6% of people with migraine had visited the GP vs. 61.0% of people without migraine (*p* < 0.001), 11.8% vs. 4.3% had visited the neurologist (*p* < 0.001), 51.4% vs. 31.2% had visited the ER (*p* < 0.001), and 17.0% vs. 8.3% had been hospitalized at least once (*p* < 0.001). The mean number of visits to the GP, neurologist and ER per person were also higher amongst migraine patients (*p* < 0.001). Mean visits to the ER in the past 6 months were more than three times higher in individuals with ≥ 15 MHD than in those without migraine (2.3 vs. 0.7 ER visits, *p* < 0.001). The difference in mean hospitalizations per patient was not statistically significant (*p* = 0.359, Table 2).

#### Direct and indirect economic burden

**Fig. 5:**
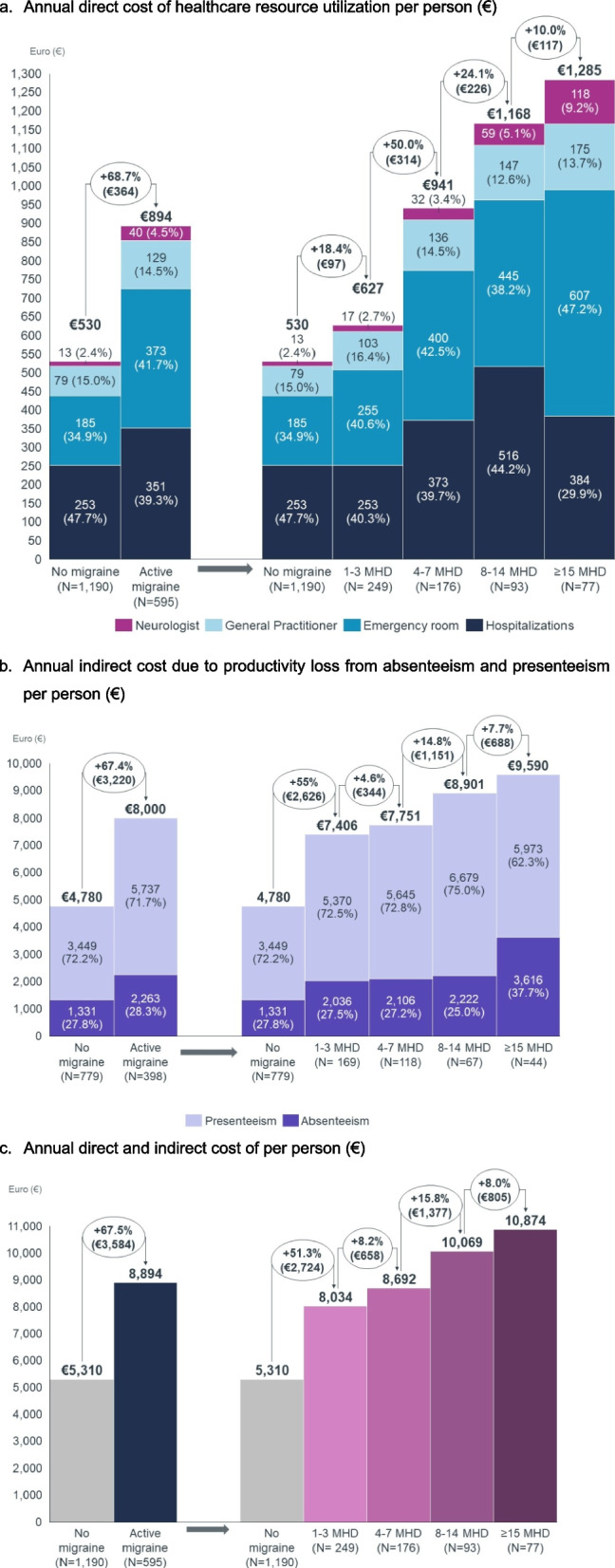
Annual direct healthcare costs and indirect costs per patient, per cohort. MHDs, monthly headache days

The annual cost per migraine patient was estimated at €8,894, of which €894 (10.1%) from direct costs and €8,000 (89.9%) from indirect costs related to absenteeism and presenteeism, whereas the annual cost per person without migraine stood at €5,310, of which €530 (10.0%) from direct healthcare costs (Fig. 5). Mean annual cost per person grew with the frequency of headaches, namely from € €8,034 in individuals with 1–3 MHD to €10,874 in individuals with ≥ 15 MHD. Regarding direct healthcare costs from HCRU, visits to the ER accounted for 41.7% of costs in the active migraine cohort (vs. 34.9% in the no-migraine cohort), followed by hospitalizations (39.3% vs. 47.7%), visits to the GP (14.5% vs. 15.0%), and visits to the neurologist (4.5% vs. 2.4%).

### Discussion

This study evaluated the societal and economic burden of migraine in Spain. As expected, in comparison with the no-migraine cohort, people who reported a physician diagnosis of migraine and had at least one migraine headache day in the preceding month, had worse quality of life; were more likely to suffer from depression; presented lower ability to work and to conduct regular daily activities; missed or were impaired at work more often; consumed more healthcare resources; and generated greater direct healthcare costs and indirect economic costs due to productivity losses.

Patients with migraine were categorized into four groups, reflecting the thresholds that are usually associated with CM (≥ 15 MHD), high-frequency episodic migraine (8–14 MHD), episodic migraine with preventive treatment indication (4–7 MHD) and episodic migraine with no need for preventive treatment (1–3 MHD) [27]. Logically, the higher the frequency of headaches the worse the outcomes were, in line with prior studies [10, 14, 28–34]. However, some of the key findings from this study also reveal an important burden of low frequency migraine, which is also the most common. These results should be taken into account when considering the use of preventive treatments, as there is an evident need for effective preventive treatments that are able to reduce the MHD and mitigate the burden of migraine across all spectrum of headache frequency [35, 36]. A recent multi-country study, including Spanish sites, has shown that migraine patients who improved over a 6-month period after initiating preventive migraine treatment had significantly lower migraine-related HCRU and costs than patients with stable or worsened migraine [36].

In this study, the annual cost per migraine patient was estimated at €8,894, of which €894 (10.1%) were from direct costs – extrapolating the HCRU values reported for the past 6 months to a complete year -, and €8,000 (89.9%) were from indirect costs related to absenteeism and presenteeism. Suffering from 1–3 MHD resulted in an additional annual cost of € 2,724 per person in comparison with people without migraine (1.5 higher). In patients experiencing ≥ 15 MHD, the cost doubled the one from those without migraine. Yet, the real direct cost of migraine is expected to be superior to the values obtained from this study, as only visits to the GP, neurologist, ER and hospitalizations were accounted for, leaving out costs with medication, visits to other specialists/ healthcare professionals, and diagnostic tests (e.g., magnetic resonance imaging, computerized tomography, lumbar puncture). Indeed, our estimates are lower than others reported for Spain, particularly in the direct healthcare costs [3, 10, 13]. Caronna et al. (2021), using a cohort of employees with migraine working in a Spanish tertiary hospital, estimated an annual cost/person of €11,112, of which €3,436 (30.9%) were from HCRU and €7,675 (69.1%) were from indirect costs related to absenteeism and presenteeism [13]. The 2018 Spanish Atlas of migraine estimated total annual costs per migraine patient of (€5,041 for EM and €12,970 for CM, of which €7,465 were attributed to productivity losses (57.8%), €3,847 from direct healthcare costs (29.8%), and €1,610 from direct costs borne by patients (12.4%) [3, 10]. Differences may be partially explained by the type of HCRU considered in each study. Moreover, it is possible that HCRU may have been lower than in previous years because of the COVID-19 pandemic. To properly put this evidence in context and extrapolate our results, we not only calculated the cost difference, but the proportion of cost increase that was observed in patients with migraine, since the ultimate cost depends on the national wages and may differ from other settings.

Evidence from this study supports the importance of using MHD to stratify patients, instead of separately analyzing EM and CM [14, 29, 31, 33, 37]. Future studies could benefit from employing similar cohorts, as little is known on the burden of low frequency migraine in Europe [30]. Overall, this study shed some light on the unmet clinical and economic burden of migraine, even in patients suffering from less than 4 MHD. As most migraine patients suffer from fewer monthly headache episodes, no strategy aimed to lessen the burden of migraine—the second-highest cause of disability worldwide—will be complete without addressing the unmet needs in this population [1, 7].

#### Limitations

Importantly, as the 2020 Spanish NHWS was conducted between 30 December 2019 and 20 April 2020, results may be partially affected by the first wave of the COVID-19 pandemic in Spain. The first case of locally acquired COVID-19 in Spain was confirmed on 26 February, a national lockdown was implemented on 15 March 2020 and the epidemic peaked on 20 March [38]. In the questionnaire, respondents were asked to provide their inputs based on the preceding six months. Additionally, the overlap with the COVID-19 pandemic may help explain the high prevalence of depression in the no-migraine control group. Studies conducted in Spain, found that prevalence rates of depression changed from 3.1%, before the COVID-19 outbreak, to 12.0%, after it. The difference was higher amongst younger people (increasing from 2.6% to 18.1% in those aged 18–29 years old and from 2.7% to 14.3% in the 30–49 group) [39]. Globally, there are mixed findings regarding the impact of the COVID-19 lockdown in the clinical course of migraine [40–42]. The impact varied per country and per patient, depending on factors such as lifestyle changes, infections, changes in depression and anxiety triggers, amongst others. Some studies report improved migraine symptoms due to the increased number of sleep hours, decreased analgesic intake, and reduction of stressor coming from working activity [41, 42]. A retrospective survey study conducted in Spain, has noted an intensification of the typical pain in about half of the individuals suffering from migraines, with the deterioration in the clinical progression of migraine being associated with alterations in their usual triggers and the psychological effects of the lockdown [40]. Nonetheless, the COVID-19 lockdown was only in place during the last month of our study, thus minimizing the impact in results.

As all NHWS survey data collected in this study was self-reported it can potentially suffer from biases due to inaccurate recall and false reporting. However, the survey is naturalistic, and no incentive is given to misrepresent one’s reporting. Being based on a sample there are inherent limitations with the representativity of NHWS data. Although it is a nationally representative general population survey that uses stratified quota sampling to recruit respondents, most respondents are recruited online. Therefore, these data may not account for the representation of certain groups, including those without access to the internet. To mitigate this risk, in Spain, online panel recruitment was complemented by computer-assisted web interviews in which respondents were recruited via telephone and could choose to complete the interview on the phone, on a computer in a private center, or through an emailed link [14]. As the NHWS addresses general health, no migraine-specific questionnaires were used to measure the studied outcomes. It is possible that migraine-specific questionnaires would be more suitable to study the differences across cohorts. Direct costs may be underestimated as only a portion of medical costs were accounted for. Constant HCRU throughout the year were assumed to annualize the direct cost, assumption which may underestimate the annual HCRU as the frequency, severity, and duration of migraine attacks is reported in some studies to increase during spring [43, 44]. The economic burden itself may not be fully comparable with other countries or settings, however, the relative difference between patients with and without migraine may be.

### Conclusions

Having migraine not only causes a massive impact on patients’ quality of life and ability to work, but it also generates considerable economic costs for society. In Spain, having migraine was associated to 1.7 higher costs per patient. The clinical and economic burden increases with the frequency of headaches but is higher than controls even in patients suffering from 1–3 MHD. This study underlines important unmet needs, with more than half of migraine patients suffering from ≥ 4 MHDs, which were in turn associated with poorer outcomes. This study provides an additional reason for considering effective treatments, to mitigate the societal and economic burden of migraine.

### Supplementary Information

**Supplementary Material 1.**

## Data Availability

No datasets were generated or analysed during the current study.

## Abbreviations

BMI: Body Mass Index
CCI: Charlson Comorbidity Index
CM: Chronic migraine
EM: Episodic migraine
ER: Emergency Room
FT: Full-time
GH: General Health
GP: General Practitioner
HRQoL: Health‑Related Quality of Life
HCRU: Healthcare Resource Utilization
INE: Instituto Nacional de Estadística
MCS: Mental Component Summary
MHD: Monthly headache days
NHWS: National Health and Wellness Survey
WPAI: Work Productivity and Activity Impairment
SD: Standard Deviation
SE: Self-employed
SMD: Standardized Mean Difference
PCS: Physical Component Summary
PT: Part-time

## References

[1] Steiner T, Stovner L, Jensen R, Uluduz D, Katsarava Z (2020). Migraine remains second among the world’s causes of disability, and first among young women: findings from GBD2019. BioMed Central.

[2] Mateos V, Porta-Etessam J, Armengol-Bertolin S, Larios C, Garcia M (2012). Initial situation and approach to the care of migraine in neurology services in Spain: the PRIMERA study. Rev Neurol.

[3] Garrido Cumbrera M, Gálvez Ruiz D, Braçe O, Nieblas Rosado MI, Delgado Domínguez CJ (2018). Impacto y situación de la Migraña en España: Atlas 2018.

[4] Ruiz M, León C, Castillo J, Martínez M, Sánchez S, Quintela E (2010). Distribución por diagnósticos de las cefaleas que acuden a los servicios de urgencias de atención primaria. SEMERGEN-Medicina de Familia.

[5] (2018) Headache Classification Committee of the International Headache Society (IHS) The International Classification of Headache Disorders, 3rd edition. Cephalalgia 38(1):1–211 10.1177/0333102417738202 29368949

[6] Adams AM, Serrano D, Buse DC, Reed ML, Marske V, Fanning KM, Lipton RB (2015). The impact of chronic migraine: The Chronic Migraine Epidemiology and Outcomes (CaMEO) Study methods and baseline results. Cephalalgia.

[7] Katsarava Z, Buse DC, Manack AN, Lipton RB (2012). Defining the differences between episodic migraine and chronic migraine. Curr Pain Headache Rep.

[8] Katsarava Z, Mania M, Lampl C, Herberhold J, Steiner TJ (2018). Poor medical care for people with migraine in Europe–evidence from the Eurolight study. J Headache Pain.

[9] Linde M, Gustavsson A, Stovner LJ, Steiner TJ, Barré J, Katsarava Z, Lainez J, Lampl C, Lantéri-Minet M, Rastenyte D (2012). The cost of headache disorders in Europe: the Eurolight project. Eur J Neurol.

[10] Irimia P, Garrido-Cumbrera M, Santos-Lasaosa S, Braçe O, Colomina I, Blanch C, Pozo-Rosich P (2020). Estimating the savings associated with a migraine-free life: results from the Spanish Atlas. Eur J Neurol.

[11] Darbà J, Marsà A (2020). Analysis of the management and costs of headache disorders in Spain during the period 2011–2016: a retrospective multicentre observational study. BMJ Open.

[12] Fernández-Ferro J, Ordás-Bandera C, Rejas J, Ferro-Rey B, Gómez-Lus S (2022). EE504 the economic burden of migraine in spain: a nationwide cost-of-illness approach from the year 2020 European health survey in spain. Value in Health.

[13] Caronna E, Gallardo VJ, Alpuente A, Torres-Ferrus M, Pozo-Rosich P (2022). Epidemiology, work and economic impactof migraine in a large hospital cohort: time to raise awareness and promote sustainability. J Neurol..

[14] Buse DC, Pozo-Rosich P, Dupont-Benjamin L, Balkaran BL, Lee L, Jauregui A, Gandhi P, Parikh M, Reuter U (2023). Impact of headache frequency and preventive medication failure on quality of life, functioning, and costs among individuals with migraine across several European countries: need for effective preventive treatment. J Headache Pain.

[15] Domínguez-Ortega J, Plaza V, Li VW, Fonseca E, Cea-Calvo L, Martin A, Sánchez-Jareño M, Brady JE, Schelfhout J (2022). Prevalence of chronic cough and patient characteristics in adults in Spain: A population-based cross-sectional survey. Chron Respir Dis.

[16] Charlson ME, Pompei P, Ales KL, MacKenzie CR (1987). A new method of classifying prognostic comorbidity in longitudinal studies: development and validation. J Chronic Dis.

[17] Montazeri A, Vahdaninia M, Mousavi SJ, Asadi-Lari M, Omidvari S, Tavousi M (2011). The 12-item medical outcomes study short form health survey version 2.0 (SF-12v2): a population-based validation study from Tehran Iran. Health Qual Life Outcomes.

[18] Maruish M (2011). User’s Manual for the SF-36v2 Health Survey.

[19] Herdman M, Gudex C, Lloyd A, Janssen M, Kind P, Parkin D, Bonsel G, Badia X (2011). Development and preliminary testing of the new five-level version of EQ-5D (EQ-5D-5L). Qual Life Res.

[20] Ware JE, Keller SD, Kosinski M (1995) SF-12: How to score the SF-12 physical and mental health summary scales. Health Institute, New England Medical Center

[21] Kroenke K, Spitzer RL, Williams JB (2001). The PHQ-9: validity of a brief depression severity measure. J Gen Intern Med.

[22] Irimia P, García-Azorín D, Núñez M, Díaz-Cerezo S, de Polavieja PG, Panni T, Sicras-Navarro A, Sicras-Mainar A, Ciudad A (2022). Persistence, use of resources and costs in patients under migraine preventive treatment: the PERSEC study. J Headache Pain.

[23] INE (2023) Calculo de variaciones del Indice de Precios de Consumo (sistema IPC base 2021) In. Edited by Estadística INd

[24] INE (2021) Distribución salarial: Medias y percentiles por sexo y CCAA. In. Edited by Estadística INd

[25] Dyer M, Frieze A (1991). Randomized greedy matching. Random Struct Algorithms.

[26] Thompson SG, Barber JA (2000). How should cost data in pragmatic randomised trials be analysed?. BMJ.

[27] Olesen J (2018). International classification of headache disorders. Lancet Neurol.

[28] Blumenfeld A, Varon S, Wilcox T, Buse D, Kawata A, Manack A, Goadsby P, Lipton R (2011). Disability, HRQoL and resource use among chronic and episodic migraineurs: results from the International Burden of Migraine Study (IBMS). Cephalalgia.

[29] Buse DC, Fanning KM, Reed ML, Murray S, Dumas PK, Adams AM, Lipton RB (2019). Life with migraine: effects on relationships, career, and finances from the chronic migraine epidemiology and outcomes (CaMEO) study. Headache.

[30] Doane MJ, Gupta S, Fang J, Laflamme AK, Vo P (2020). The humanistic and economic burden of migraine in Europe: a cross-sectional survey in five countries. Neurol Therapy.

[31] Ishii R, Schwedt TJ, Dumkrieger G, Lalvani N, Craven A, Goadsby PJ, Lipton RB, Olesen J, Silberstein SD, Burish MJ (2021). Chronic versus episodic migraine: The 15-day threshold does not adequately reflect substantial differences in disability across the full spectrum of headache frequency. Headache.

[32] Vo P, Fang J, Bilitou A, Laflamme AK, Gupta S (2018). Patients’ perspective on the burden of migraine in Europe: a cross-sectional analysis of survey data in France, Germany, Italy, Spain, and the United Kingdom. J Headache Pain.

[33] Buse DC, Reed ML, Fanning KM, Bostic RC, Lipton RB (2020). Demographics, headache features, and comorbidity profiles in relation to headache frequency in people with migraine: results of the American Migraine Prevalence and Prevention (AMPP) study. Headache.

[34] Pozo-Rosich P, Lucas C, Watson DP, Gaul C, Ramsden E, Ritter S, Martelletti P, Snellman J (2021). Burden of migraine in patients with preventive treatment failure attending European headache specialist centers: Real-world evidence from the BECOME study. Pain Ther.

[35] Doane MJ, Gupta S, Vo P, Laflamme AK, Fang J (2019). Associations between headache-free days and patient-reported outcomes among migraine patients: a cross-sectional analysis of survey data in Europe. Pain Ther.

[36] Vo P, Swallow E, Wu E, Zichlin ML, Katcher N, Maier-Peuschel M, Naclerio M, Ritrovato D, Tiwari S, Joshi P (2021). Real-world migraine-related healthcare resource utilization and costs associated with improved vs. worsened/stable migraine: a panel-based chart review in France, Germany, Italy, and Spain. J Med Econ.

[37] Torres-Ferrús M, Quintana M, Fernandez-Morales J, Alvarez-Sabin J, Pozo-Rosich P (2017). When does chronic migraine strike? A clinical comparison of migraine according to the headache days suffered per month. Cephalalgia.

[38] Working group for the surveillance and control of COVID-19 in Spain (2020) The first wave of the COVID-19 pandemic in Spain: characterisation of cases and risk factors for severe outcomes, as at 27 April 2020. Euro Surveill. 25(50):pii=2001431. 10.2807/1560-7917.ES.2020.25.50.2001431 10.2807/1560-7917.ES.2020.25.50.2001431 PMC7812423 33334400

[39] Ayuso-Mateos JL, Morillo D, Haro JM, Olaya B, Lara E, Miret M (2023). Changes on depression and suicidal ideation under severe lockdown restrictions during the first wave of the COVID-19 pandemic in Spain: a longitudinal study in the general population. Epidemiol Psychiatr Sci.

[40] Gonzalez-Martinez A, Planchuelo-Gómez Á, Guerrero ÁL, García-Azorín D, Santos-Lasaosa S, Navarro-Pérez MP, Odriozola-González P, Irurtia MJ, Quintas S, de Luis-García R (2021). Evaluation of the Impact of the COVID-19 Lockdown in the Clinical Course of Migraine. Pain Med.

[41] Parodi IC, Poeta MG, Assini A, Schirinzi E, Del Sette P (2020). Impact of quarantine due to COVID infection on migraine: a survey in Genova Italy. Neurol Sci.

[42] Reyes-Alvarez MT, Bancalari E, Santana Vargas AD, Velez K, Rodríguez-Leyva I, Marfil A, Miranda S, Zegarra-Valdivia JA (2023). Impact of COVID-19 Pandemic Lockdown on Migraine Patients in Latin America. Int J Environ Res Public Health.

[43] Brewerton TD, George MS (1990). A study of the seasonal variation of migraine. Headache.

[44] Robbins L (1994). Precipitating factors in migraine: a retrospective review of 494 patients. Headache.

