# Supplementary material for: Societal and economic burden of migraine in Spain: results from the 2020 National Health and Wellness Survey
Source: J Headache Pain. 2024 Mar 15;25(1):38. doi: 10.1186/s10194-024-01740-3 (PMC10941425; doi:10.1186/s10194-024-01740-3)
Supplement: Supplementary file 1 — Supplementary Material 1. [file 10194_2024_1740_MOESM1_ESM.docx]

### S1. List of study variables and definitions

***Demographic characteristics***

Demographics of interest included: age, sex (male or female), country of residence (France, Germany, UK, Italty, Spain), marital status (married or living with partner or not), household income (low, medium, high, or refuse to answer), level of education (completed university education or not), employment status (yes or no).

***Health and clinical characteristics***

MHD were grouped by frequencies of 1-3, 4-7, 8-14, and 15+ days in the past 30 days for those with migraines and were compared in aggregate and by each group with matched non-migraine comparators.

Other health characteristics of interest included smoking status (current, former, or never), alcohol use (yes or no), vigorous exercise in past 30 days (yes or no), body mass index (BMI; underweight, normal weight, overweight, obese, or decline to answer).

The Charlson Comorbidity Index (CCI) was assessed as a measure of overall comorbidity burden.

Cardiovascular diseases and risk factors were also assessed. Prevalence of patient-reported diagnoses of angina, arrhythmia, atherosclerosis, atrial fibrillation, congestive heart failure, heart attack, high blood pressure, high cholesterol, left ventricular hypertrophy, transient ischemic attack, peripheral artery disease, peripheral vascular disease, stroke, and unstable angina. A combined cardiovascular disease prevalence included all conditions. An additional severe cardiovascular disease measure included: angina, arrhythmia, atherosclerosis, atrial fibrillation, congestive heart failure, heart attack, left ventricular hypertrophy, transient ischemic attack, peripheral artery disease, peripheral vascular disease, stroke, and unstable angina. Additional descriptions included any cardiovascular risk factor included 1 or more and 2 or more were also reported (any of the above plus current smoking status and obesity).

***Healthcare resource utilization***

Respondents were asked about HCRU in the past 6 months. This included overall office-based healthcare visits and physician specialties (e.g., family medicine, neurologist, etc.) and other healthcare providers (e.g., nurse practitioners). Emergency room (ER) visits and hospitalizations were recorded. HCRU was summarized by any utilization (yes or no) as well as the mean number of visits for each provider type and care setting in the past 6 months.

***Quality of life***

QoL was assessed using two validated scales: 1) the Medical Outomes Study Short Form Health Survey 12, version 2 (SF-12) and 2) the EuroQol 5 Dimensions (EQ-5D) index score. The SF-12 included a 4-week recall period to assess generic health status using 12 questions. A mental (MCS) and physical (PCS) component score, ranging from 0 to 100, are calculated with higher values indicating better overall health. The EQ-5D is a preference-based measure of health ranging from 0 to 1 (1 equal to full health and 0 equal to death). The EQ-5D index score is calculated from standard value sets for each country. The EQ-5D includes a visual analog scale (EQ-VAS) where respondents indicate their health ranging from 0 to 100 (best possible health).

Prior studies observed increased mental health conditions in people with migraine, thus the prevalence and severity of depression was also assessed. The Patient Health Questionnaire 9 item (PHQ-9) is a validated depression module that assesses a 2-week recall for 9 diagnostic criteria ranging from no occurrence to “nearly every day.” The PHQ-9 provides a summary score and categorizes people based on no or minimal, mild, moderate, moderately severe, and severe depression.

***Work productivity and activity impairment***

Work productivity and activity impairment (WPAI) were assessed using the General Health version of the WPAI questionnaire. The WPAI health version is a validated 6-item instrument that measures 4 metrics, including absenteeism (absence from work due to health), presenteeism (present at work but not fully functional due to health), overall work productivity loss, and activity impairment. Measures are recorded as percentages of impairment, with higher values indicating greater impairment and less productivity. Respondents who reported any employment provided data for absenteeism, presenteeism, and overall work impairment. All respondents completed the activity impairment items, which includes activity limitations outside of employment.

### S2. Unit costs per HCRU

| **Unit** | **Unit cost 2019** | **Unit cost 2023 prices** |
| --- | --- | --- |
| Primary care visit | € 23.2 | € 26.5 |
| Specialist (neurologist) visit | € 92.5 | € 105.6 |
| Emergency room visit | € 117.5 | € 134.2 |
| Hospitalization | € 480.9 | € 549.2 |

### S3. Input data used to compute the estimated annual indirect cost per person

|  | **No Migraine** | **Migraine** | **1-3 MHD** | **4-7 MHD** | **8-14 MHD** | **≥15 MHD** |
| --- | --- | --- | --- | --- | --- | --- |
| **Estimated mean annual household income (€)** | € 27,006 | € 27,072 | | | | |
| **Work productivity Impairment (%)** | 25.53 | 41.37 | 38.30 | 40.08 | 46.03 | 49.59 |
| **Annual Work productivity loss cost per person (€, unweighted)** | € 6,895 | € 11,200 | € 10,369 | € 10,851 | € 12,461 | € 13,425 |
| **Employed population (%)** | 69.33 | 71.43 | | | | |
| **Annual Work productivity loss cost per person (€, weighted)** | € 4,780 | € 8,000 | € 7,406 | € 7,751 | € 8,901 | € 8,590 |
